# The Effects of 810 nm Diode Laser and Indocyanine Green on Periodontal Parameters and HbA1c in Patients with Periodontitis and Type II Diabetes Mellitus: A Randomized Controlled Study

**DOI:** 10.3390/diagnostics12071614

**Published:** 2022-07-02

**Authors:** Irina-Georgeta Sufaru, Maria-Alexandra Martu, Ionut Luchian, Simona Stoleriu, Diana Diaconu-Popa, Cristian Martu, Silvia Teslaru, Liliana Pasarin, Sorina Mihaela Solomon

**Affiliations:** 1 Department of Periodontology, Grigore T. Popa University of Medicine and Pharmacy, Universitatii Street 16, 700115 Iasi, Romania; ursarescu.irina@umfiasi.ro (I.-G.S.); silvia.teslaru@umfiasi.ro (S.T.); liliana.pasarin@umfiasi.ro (L.P.); sorina.solomon@umfiasi.ro (S.M.S.); 2 Department of Cariology and Restorative Dental Therapy, Grigore T. Popa University of Medicine and Pharmacy, Universitatii Street 16, 700115 Iasi, Romania; simona.stoleriu@umfiasi.ro; 3 Department of Oral Implantology, Removable Dentures and Technology, Grigore T. Popa University of Medicine and Pharmacy, Universitatii Street 16, 700115 Iasi, Romania; antonela.diaconu@umfiasi.ro; 4 ENT Clinic Department, Grigore T. Popa University of Medicine and Pharmacy, Universitatii Street 16, 700115 Iasi, Romania; cristimartu@gmail.com

**Keywords:** diode laser, indocyanine green, periodontitis, type II diabetes mellitus, clinical periodontal parameters, HbA1c

## Abstract

The aim of this study was to investigate the effects of adjunctive periodontal therapy of 5 mg/mL indocyanine green irradiation by an 810 nm diode laser (aPDT), supplementary to scaling and root planing (SRP) in patients with periodontitis and type II diabetes mellitus (DM) compared to the SRP alone, on periodontal clinical parameters and glycated hemoglobin A1c (HbA1c). The study was conducted on 49 subjects with type II DM and periodontitis, divided into two groups: the SRP group (n = 25), who followed SRP alone, and the SRP + aPDT group (n = 24), who followed SRP and four weekly sessions of indocyanine green irradiation by an 810 nm diode laser. Plaque Index (PI), Bleeding on Probing Index (BOP), probing depth (PD), clinical attachment loss (CAL) and HbA1c were investigated at baseline and after 6 months. At 6 months, both SRP alone and SRP + aPDT generated significant reductions in all the investigated parameters; SRP + aPDT produced more significant reductions for BOP, PD and CAL (*p* < 0.001) but not for PI and HbA1c, than SRP alone. aPDT with indocyanine green therapy was well tolerated, with two subjects reporting slight discomfort. Therefore, aPDT with indocyanine green might represent a good adjunctive periodontal treatment option for SRP in patients with type II DM and periodontitis.

## 1. Introduction

Periodontitis is an inflammatory disease of the tissues that serve to maintain and sustain the functionality of the teeth on the dental arches. Its etiology is often multifactorial, the main cause being the onset of periodontal dysbiosis, in favor of anaerobic gram-negative bacteria [1]. The human body will react to this dysbiosis through innate and adaptive defense mechanisms, with the manifestation of an inflammatory response, detectable at molecular and clinical levels. If the causal factors are not removed, the inflammation may evolve, turning initially reversible lesions into attachment losses, with the onset of periodontitis [2].

A number of local and systemic factors can influence either the retention of periodontopathogenic bacterial plaque, with changes in its quantity and quality, or the ability of the immune system to effectively counteract bacterial aggression [3]. The latter include diabetes mellitus (DM). This study focuses on type II DM patients with periodontitis; type II DM is a metabolic disease, often the consequence of an inadequate diet, which may be associated with other predisposing factors (tobacco use, alcohol, genetic factors, etc.) [4]. The overall impact of DM is epidemic in nature, with worrying statistical parameters, especially in light of the complications that patients with diabetes may experience. These include changes of a micro- and macro-vascular nature, which make DM the leading cause of blindness (diabetic retinopathy), amputation of the lower limbs, kidney failure, hypertension or even death from cardiovascular causes [5]. Periodontal disease has been identified as the sixth complication of DM, as a result of a combination of mechanisms that include reduced immune response capacity in the patient with diabetes, increased markers of inflammatory and oxidative stress through the production of advanced glycation end-products (AGEs) and tissue healing deficits [6]. Of course, the manifestations of these complications are all the more important as the metabolic control is more deficient [7]. A measure of this ability is the evaluation of glycated hemoglobin (HbA1c). Moreover, it seems that periodontal inflammatory status can also negatively influence metabolic control in patients with diabetes [8].

The treatment of the patient with periodontal impairment includes, first of all, the removal of the risk factors, which, in the first stage, involves, in addition to the patient’s motivation and awareness, professional hygiene techniques [9]. Scaling and root planing (SRP) remain the gold standard in periodontal therapy, with the aim of obtaining “plaque-free” coronal and root surfaces, with a relief that allows the creation of a new periodontal attachment. Nevertheless, the majority of clinical trials have suggested that non-surgical periodontal debridement improves glycemic control among individuals with type 2 diabetes mellitus [10,11,12]. A meta-analysis performed by Jain et al. [12] found that SRP treatment decreased HbA1c by 0.26% (*p* = 0.17) at 3–4 months compared to the control group. A systematic review and meta-analysis [13] found that SRP generated a statistically significant reduction in HbA1C levels at 3 months of about 0.40% (range 0.27–0.65%).

However, it seems that SRP cannot completely remove bacterial species and their products, especially from periodontal soft tissues [14]. Thus, new methods of additional periodontal treatment have been developed, methods that involve local and/or systemic administration of antiseptic or antibiotic substances [15], therapies to modulate the host immune response (sub-antimicrobial doses of doxycycline) [16] or photo-bio-modulation therapies and photo-disinfection of periodontal pockets [17].

Antimicrobial photodynamic therapy (aPDT) is an adjuvant, minimally invasive therapeutic method that involves the use of three main components: light source (laser or LED), photosensitizer and singlet oxygen released into the tissue, the latter generating the bactericidal effect [18]. Most studies investigating the effects of aPDT in patients with periodontitis have used phenothiazine derivatives (methylene blue, toluidine blue), xanthene or riboflavin as photosensitizers. Recently, special attention has been paid to indocyanine green, as a product with photosensitizing potential [17].

Indocyanine green consists of two aromatic parts linked together by a polyunsaturated chain; it is a dye commonly used in medicine, especially in imaging investigations [19]. It penetrates rapidly into tissues and has low toxicity, being approved by the FDA for clinical use and recognized as non-toxic [20]. The absorption range for indocyanine green is between 600 nm and 900 nm and it emits fluorescence between 750 nm and 950 nm [21].

Data on the efficacy of this substance in patients with periodontitis and DM are limited; for this reason, we propose a study aimed at investigating the effects on periodontal clinical parameters and HbA1c of aPDT adjunctive therapy with indocyanine green as a photosensitizer in patients with periodontitis and type II diabetes, compared to the use of SRP alone. Our primary outcome investigation regarded the local, periodontal response to treatment of a particular group of subjects, prone to severe periodontal destructions and impaired healing. As a secondary outcome, we wanted to assess any potential supplementary benefits other than those already demonstrated by scaling and root planing, on HbA1c.

The proposed null hypothesis was that aPDT with indocyanine green does not provide any additional benefits to SRP on local periodontal parameters, nor on glycemic control, measured by HbA1c, for this particular category of patients.

## 2. Materials and Methods

### 2.1. Patient Selection

This prospective, randomized controlled, single-blind interventional study was performed on 49 patients diagnosed with periodontitis and type II diabetes. The methodology of the study was in accordance with the rules set out in the Declaration of Helsinki and was approved by the Bioethics Commission of the institution. All study participants signed the informed consent form and were aware of their right to withdraw from the study at any stage of the study without being subject to any sanctions.

The study included male and female subjects, diagnosed with type II diabetes mellitus by the diabetologist, and who presented periodontal probing depths higher than 5 mm upon periodontal examination. Exclusion criteria were represented by: (i) systemic diseases other than diabetes, which could influence periodontal status; (ii) smoking; (iii) history of antibiotic therapy, anti-inflammatory therapy or periodontal treatment in the last 3 months; (iv) significant changes in DM treatment during the study period.

### 2.2. Sample Size Calculation and Randomization

Based on previous findings [22], a probing depth (PD) reduction of 1 mm was used to determine the size of the groups, with a power of 90%, and alpha set to 0.05, resulting in an optimal size of 22 subjects per group. However, in order to counteract potential withdrawals from the study, we set a size of 25 patients per group, estimating an abandonment rate of 10%. The 50 resulting subjects were, thus, divided into two groups: patients who followed scaling and root planing therapy (n = 25) (SRP group) and patients who followed SRP and aPDT therapy with diode laser and indocyanine green (n = 25) (SRP + aPDT group); one patient who did not attend all aPDT sessions was finally excluded from the study group.

The SRP group consisted of 56.00% male subjects and 44.00% female subjects, with an overall mean age of 54.24 ± 3.41 years old; the SRP + aPDT group consisted of 54.20% male subjects and 45.80% female subjects, with an overall mean age of 55.58 ± 3.62 years old. There were no significant differences in age between the two groups (*p* = 0.734) (Table 1).

The distribution of patients to one of the two groups was randomized, through a system of sealed envelopes. Participants were offered randomly generated treatment assignments in sealed opaque envelopes, doubled with carbon paper, the therapeutic operator not being involved in making the envelopes.

### 2.3. Clinical Investigations

For each patient included in the study, the following periodontal parameters were determined: (a) the simplified plaque index (PI) [23], with the qualitative percentage determination of the surfaces with bacterial plaque; (b) bleeding on probing index (BOP), with the qualitative determination of the percentage of bleeding sites following the periodontal probing; (c) probing depth (PD), established by inserting the periodontal probe into the periodontal pocket, evaluated at six points per tooth (mesial–buccal, buccal, distal–buccal, mesial–lingual, lingual, distal–lingual); (d) periodontal clinical attachment loss (CAL), assessed as the distance between the enamel–cement junction and the base of the periodontal pocket. A North Carolina No.15 Periodontal Probe (Hu-Friedy, Chicago, IL, USA) was used for clinical measurements.

All periodontal assessments were performed by an experienced qualified examiner. Calibration was accepted if >90% of the measurements were reproduced within 48 h. Clinical examinations were performed blind to the type of treatment followed by the subjects. Clinical measurements were performed at baseline (T0) and 6 months apart (T1).

### 2.4. Evaluation of HbA1c

For the analysis of glycated hemoglobin, venous blood was collected in 3 mL vacutainer with EDTA K3 (FL Medical, Torreglia PD, Italy). The quantification of glycated hemoglobin in total hemolyzed blood was based on a turbidimetric inhibition reaction, as previously described [24,25]. Glycated hemoglobin A1c (HbA1c) was determined for each patient at baseline and after 6 months. The method of determining HbA1c was immunoturbidimetric (Boehringer Mannheim, Baden-Wurttemberg, Germany), a test characterized by high specificity for anti-HbA1c antibodies [26].

### 2.5. Treatment Methods

All patients in the SRP and SRP + aPDT groups underwent non-surgical periodontal treatment, which involved ultrasound scaling (Woodpecker UDS-A-LED, Guilin Woodpecker Medical Instrument Co., Ltd., Guangxi, China) and root planing (Gracey Standard and Mini—Hu-Friedy, Chicago, IL, USA) (SRP), in one session. All patients were instructed in the technique of brushing their teeth and to avoid rinsing with antiseptics during the study.

Patients in the study group also received aPDT therapy with diode laser and indocyanine green. For the preparation of the photosensitizer, the indocyanine green powder (ICG Pulsion, PULSION Medical Systems SE, Feldkirchen, Germany) was mixed with pure water, with a ratio of 5 mL water to 25 g bottle of powder, to obtain a concentration of 5 mg/mL according to the manufacturer’s instructions. Since the aqueous solution of indocyanine green is unstable and must be used within 24 h, the fresh solution was prepared whenever necessary and the excess was discarded.

The laser system used in the present study was a diode laser (A.R.C. Laser GmbH, Nuremberg, Germany) with a wavelength of 810 nm. Based on previous findings [27,28], the laser was applied continuously with a power of 0.2 W and a total energy of 12 J. In the periodontal pocket 1 mL of indocyanine green solution was inserted with a blunt needle and left in place for 60 s. The pocket was immediately irradiated with a diode laser by placing the tip in the pocket and moving it circumferentially around the tooth for 60 s. The procedure was repeated at 7, 14 and 21 days.

**Figure 1:**
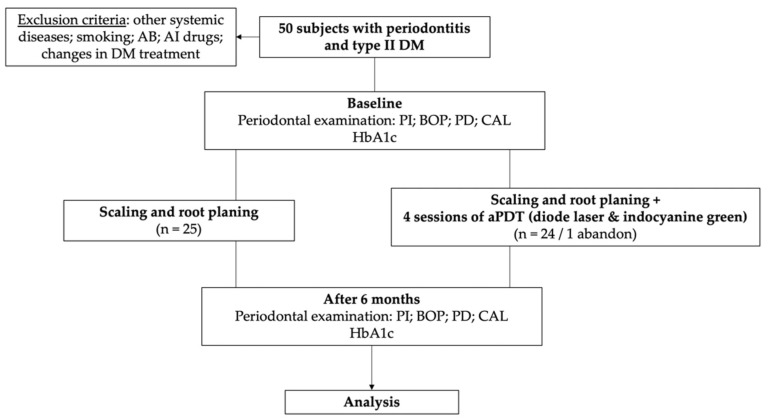
The flowchart of the study.

All the patients were instructed to follow the nutritional recommendations and to continue their normal physical activity throughout the study. The flowchart of the study from enrollment to completion is presented in Figure 1.

### 2.6. Statistical Analysis

All data were recorded in individual patient records, stored and statistically analyzed. For statistical analysis we used Microsoft Excel 2021 software (Microsoft, Washington, DC, USA) and Wizard 2 for Mac (Evan Miller^®^). The Shapiro–Wilk test was performed to determine the normality of the data distribution. The normally distributed values were compared with the *t*-Test and for the abnormally distributed values we used the Mann–Whitney test. The significance level was set at *p* < 0.05. The Pearson correlation test was used to determine the relationship between clinical parameters and HbA1c.

## 3. Results

The present study was completed in a SRP group of 25 subjects (474 sites, with a mean of 18.96 ± 4.48) who followed only SRP and a SRP + aPDT group of 24 subjects (445 sites, with a mean of 18.54 ± 4.07) that followed SRP + aPDT (Table 1).

At baseline, the PI values in the SRP group were 79.44 ± 6.31 and 80.04 ± 5.90 for the SRP + aPDT group, with no statistically significant difference (*p* = 0.732). After 6 months, PI showed significant decreases for both the SRP and the SRP + aPDT groups (17.72 ± 6.38, *p* < 0.001 and 17.08 ± 5.14, *p* < 0.001, respectively), with no statistically significant difference between groups at +6 months either (Table 2).

For BOP, the values at baseline were of 67.76 ± 6.57 in the SRP alone subjects and 68.67 ± 6.10 in the SRP + aPDT subjects, with a *p* = 0.620. The assessments after 6 months showed significant decreases for both groups: 8.08 ± 5.09 for the SRP group (*p* < 0.001) and 4.21 ± 3.85 for the SRP + aPDT group (*p* < 0.001); moreover, the decrease was more significant for the SRP + aPDT subjects (*p* < 0.001) (Table 2).

PD and CAL followed the same trend as BOP. At baseline, the PD and CAL values for the SRP group were of 5.54 ± 0.24 mm and 4.51 ± 0.20 mm, respectively; the values in the SRP + aPDT group were of 5.53 ± 0.24 mm and 4.50 ± 0.22 mm, with no statistically significant differences between the groups (*p* = 0.878 and *p* = 0.951, respectively). After 6 months, both parameters showed significant decreases; for the SRP group PD = 4.10 ± 0.22 mm, *p* < 0.001 and CAL = 3.15 ± 0.17 mm, *p* < 0.001; for the SRP + aPDT group, PD = 3.56 ± 0.19 mm, *p* < 0.001 and CAL = 2.58 ± 0.19 mm, *p* < 0.001. The decreases for PD and CAL were more significant in the subjects with SRP + aPDT (both *p*-values were lower than 0.001) (Table 2).

**Figure 2:**
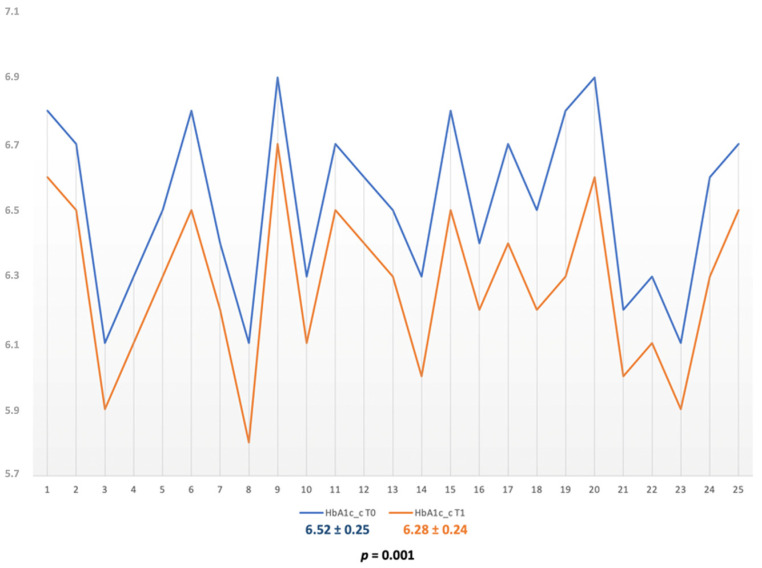
HbA1c variations in the SRP group.

**Figure 3:**
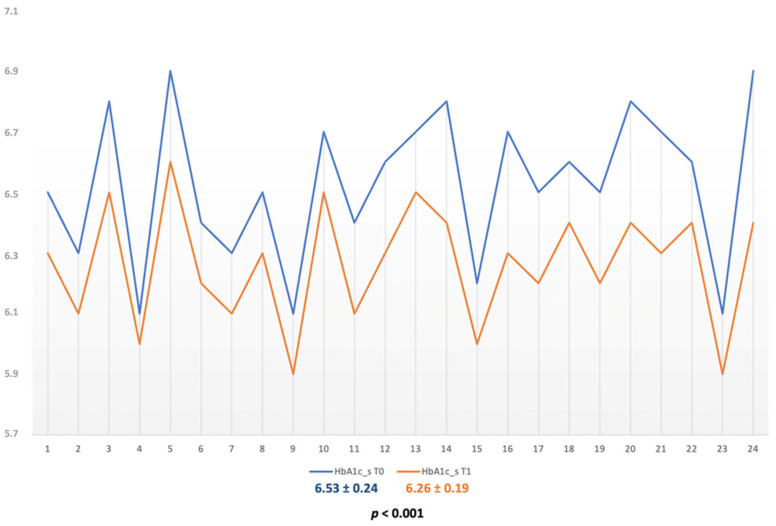
HbA1c variations in the SRP + aPDT group.

The values of HbA1c at baseline were of 6.52 ± 0.25 for the SRP group subjects and of 6.53 ± 0.24 for the SRP + aPDT group (*p* = 0.900). Both treatment options generated significant and similar reductions of HbA1c for both groups. The HbA1c value for SRP group subjects after 6 months was 6.28 ± 0.24, *p* = 0.001 (Figure 2); the value for the SRP + aPDT group was 6.262 ± 0.040, *p* < 0.001 (Figure 3) and Δ values of 0.244 ± 0.014 for the SRP alone and of 0.26 ± 0.19 for the SRP + aPDT.

We found a strong positive correlation between HbA1c and tissue loss parameters (PD and CAL) in both groups. In the SRP group, we observed a ρ = 0.922 and ρ = 0.896 for PD at baseline and +6 months, respectively; for CAL the values were ρ = 0.796 and ρ = 0.809; also, the decrease in HbA1c (ΔHbA1c) correlated with ΔPD and ΔCAL (ρ = 0.773 and ρ = 0.491, respectively). In the SRP + aPDT group, the coefficients were ρ = 0.989 and ρ = 0.995 for PD at baseline and +6 months, respectively; for CAL the values were ρ = 0.918 and ρ = 0.794; also, the decrease in HbA1c (ΔHbA1c) correlated with ΔPD and ΔCAL (ρ = 0.963 and ρ = 0.697, respectively).

The treatment regime was well tolerated, without any significant side effects. Two patients in the SRP + aPDT group (8.33%) reported slight discomfort due to the local heating during aPDT.

## 4. Discussion

The present research proposed an evaluation of the effects of an 810 nm wavelength diode laser and indocyanine green supplementary therapy on periodontal clinical parameters and HbA1c, compared to SRP alone, in patients with diabetes mellitus and periodontitis. As far as we know, this is one of the first studies to investigate the effects of indocyanine green aPDT in patients with DM.

Photodynamic therapy has emerged as an additional method for periodontal treatment, an alternative to antibiotic therapy, which can cause a number of side effects, such as, in addition to toxicity, the emergence of resistant microorganisms. Existing data in the literature on the effectiveness of aPDT with various photosensitizers are heterogeneous. While some results suggest that the benefits of SRP supplementation with aPDT are minor [29], other information, focused on the effects of aPDT with indocyanine green, would indicate that indocyanine green is more effective than previously analyzed agents in the management of periodontitis [30]. Although the local effects of using methylene blue or toluidine have been beneficial in terms of periodontal status improvements in patients with DM, they may generate a number of less desirable effects, such as tooth staining due to their prolonged adhesive property [31].

The adjunct aPDT therapy, with various photosensitizers, was previously investigated in DM subjects; the data suggest that, even if the clinical periodontal improvements were significant when compared to SRP alone, the HbA1c reduction as additional benefit were modest. Nevertheless, the found studies [32,33,34,35] had significant heterogeneity regarding the energy source, the used laser power, irradiation sessions and also concentrations for photosensitizers.

Indocyanine green is considered a safe substance without the side effects of antibiotics [17]. Mechanisms that could explain the beneficial effects of indocyanine green as an adjunct to aPDT have been investigated in clinical, microbiological, and immunological studies [27,28]. The hypotheses investigated include the bactericidal effect on periodontal pathogens, as well as potential effects on the local immune response [30].

The mechanism of action of indocyanine green is different from other photosensitizers—it exhibits a 20% photodynamic effect and the main action is through photothermal effect, which induce cell damage by increasing intracellular temperature [36]. Photothermal therapy involves the energy absorption from laser radiation by indocyanine green, inducing an effectively elevated local temperature [37]. 

In vitro studies showed that indocyanine green aPDT could effectively reduce bacterial load in periodontal pockets [31,38]. Boehm and Ciancio [38] demonstrated that indocyanine green aPDT generated a significant killing of *A. actinomycetemcomitans* and *P. gingivalis*. Srikant et al. [39] observed substantial reduction in the proportion of viable bacteria at the end of 1 week in sites receiving indocyanine green (5 mg/mL) aPDT compared to sites which followed SRP alone or SRP plus low-level laser therapy.

An investigation found that, in addition to photothermic effects, indocyanine green was shown to have a photodynamic effect by generating reactive oxygen species (ROS) [40]. Since pathogens indicate various susceptibility for singlet oxygen and radical species, it is important for future studies to measure the ROS under the selected experimental conditions. This would lead to understanding the photoreaction mechanisms and, consequently, mechanism of the applied aPDT.

Our study investigated the effects of additional aPDT with indocyanine green to SRP versus SRP alone on periodontal clinical parameters in patients with type II diabetes and periodontitis: bacterial plaque index (PI), probing bleeding index (BOP), probing depth (PD) and loss of periodontal clinical attachment (CAL), along with HbA1c analysis, assessments performed at baseline and after six months. It is important to note that there were no statistically significant differences between the groups regarding all these parameters at baseline.

At the six-month evaluations, we noticed statistically significant differences compared to baseline for all clinical parameters in both groups. Following comparisons between groups at six months, we noticed that there were no significant differences between PI values, unlike the studies conducted by Sethi and Raut [28] or Vangipuram et al. [41], where PI was significantly lower in the SRP + aPDT group than in the SRP group. We specify that in the study there was a rigorous protocol of motivation and awareness of the patient, the brushing technique being clearly explained, with the reinstatement of the instruction whenever necessary. We also cannot ignore the fact that patients may have shown high compliance as a result of awareness of participation in a study (potential Hawthorne effect) [42]. We also recommended that patients avoid oral rinses with antiseptics during the study to avoid the risk of bias.

Moreover, in a systematic review and meta-analysis it was suggested that multiple applications of aPDT are more efficient in reducing periodontal pathogens compared to a single application [43]. Based on these findings, our treatment protocol involved four sessions of aPDT.

Statistically significant differences between groups appeared when comparing BOP, PD and CAL; subjects who followed SRP + aPDT showed more significant decreases than subjects who followed only scaling and root planing. These data are consistent with the results of other studies. Al-Momani [44], in a split-mouth design study in patients with type II diabetes and stage III periodontitis, grade C, noticed significant improvements in clinical and antimicrobial parameters. Raut et al. [27] also observed a reduction in PD and CAL in the test group with indocyanine green aPDT therapy compared to the control group after 6 months. A recent meta-analysis [30] observed statistically significant improvements in aPDT results with indocyanine green at 3 months and 6 months after therapy, compared with single SRP; PD demonstrated an average additional reduction of 1.17 mm and 1.06 mm at 3 and 6 months, respectively. For CAL, an average additional gain of 0.70 mm and 1.03 mm was observed at 3 and 6 months, respectively [30].

Highly significant reductions for the indocyanine green aPDT group were also observed for BOP and PD by Monzavi et al. [36] and Hill et al. [45], without significant benefits, however, for CAL. It is worth noting, however, that they used a concentration of indocyanine green solution of 1 mg/mL. Similar to our study, Shingnapurkar et al. [46], using the same concentration of 5 mg/mL indocyanine green, observed significant reductions for CAL, in addition to BOP and PD.

Bassir et al. [47], in a study on aPDT with indocyanine green 1 mg/mL, for 30 s per session, for four sessions in patients with periodontitis, concluded that indocyanine green with an 810 nm diode laser in combination with SRP led to complete resolution of inflammation and significant reduction in periodontal pocket.

One study compared the antimicrobial efficacy of aPDT with indocyanine green, metronidazole gel, and chlorhexidine gel in vitro and reported that all of these modalities significantly reduced bacterial load [48]. However, the major clinical disadvantages associated with chlorhexidine, including taste change and pigmentation of teeth and mucosa, were absent in the case of aPDT. In addition, Chiang et al. [49] demonstrated that cytotoxicity on oral cells by aPDT with indocyanine green was significantly less prominent compared to that of chlorhexidine.

Moreover, the subgingival environment is characterized by lack of oxygen, which may not provide favorable conditions for better action of these traditional photosensitizers, while indocyanine green works even in the absence of oxygen [31]. Importantly, indocyanine green gets stimulated only in the presence of laser light, hence only the target (bacterial cells) gets affected in a dose-controlled fashion.

Thus, there is a need to establish a standard protocol for the use of indocyanine green solution in periodontal therapy of aPDT, in terms of solution concentration, but also in the number of aPDT sessions. During the studies, including the present study, which used four sessions of aPDT, no adverse effects were reported to contraindicate the repetitive application of aPDT with indocyanine green. Two patients reported mild discomfort during aPDT application but did not require discontinuation of therapy.

HbA1c followed significant decreases in both subjects who had only scaling and root planing, and those with SRP + aPDT (0.244 ± 0.014 for the SRP alone and 0.267 ± 0.02 for the SRP + aPDT). This aspect is consistent with other data in the literature—Jain et al. [12] observed, in a meta-analysis, a decrease in HbA1c by 0.26% (*p* = 0.17) at 3–4 months after SRP. This reduction can be considered, from a global point of view, as minor; further investigations, with full apprehension of all patient variables, are required in order to determine if this reduction can be based on the effects of the local, periodontal treatment. Nevertheless, even if minor, reductions of HbA1c can exert a considerable impact on the systemic status of the patient with type II diabetes, especially on its complications. Each 1% reduction in the mean HbA1c was associated with a 21% reduction in risk for diabetes-related deaths, 14% for myocardial infarction and 37% for microvascular complications [50]. What we noticed, however, in this study is that aPDT did not generate more significant effects on HbA1c than SRP alone.

Thus, we can note that the null hypothesis was partially refuted; aPDT with indocyanine green had more statistically significant effects on BOP, PD and CAL, but not on PI and HbA1c. Therefore, this type of adjunctive therapy has the potential to generate supplementary clinical benefits to SRP on the periodontal destructive status of patients with type II diabetes, with no significant benefit to SRP in terms of glycemic control.

Of course, this study also has a number of limitations. Further investigations of our proposed therapeutic protocol on larger groups of DM and periodontitis subjects are required, with the inclusion of systemically healthy subjects as controls. We also did not assess the duration of diabetes illness, a factor which can negatively impact the evolution of periodontitis and the response to periodontal treatment [51]. Even if the exact DM treatment variables were not investigated in detail, any changes in DM treatment or diet were considered as exclusion criteria.

Moreover, our study was predominantly clinical; we intend to continue and expand our investigations of the microbiological and molecular changes that could be generated by aPDT periodontal adjunctive treatment with indocyanine green in type II DM patients. Interesting observations might also emerge from comparative studies with other available photosensitizers, such as methylene blue, toluidine blue or curcumin. Moreover, further research could investigate the potential effects of this particular therapy in patients with other systemic conditions, such as osteo-articular, renal or cardiovascular diseases and periodontitis.

## 5. Conclusions

Within the limitations of our study, the therapeutic protocol of four sessions with an 810 nm wavelength diode laser and 5 mg/mL indocyanine green as adjunctive to scaling and root planing resulted in statistically higher reductions in bleeding on probing, probing depth, and periodontal clinical attachment loss in patients with type II diabetes mellitus and periodontitis, when compared to SRP alone. Further investigations need to clarify the clinical and molecular advantages of using a photosensitizer that does not require the presence of oxygen in the microaerophilic deep periodontal pockets of DM patients.

## Figures and Tables

**Table 1:** Demographic parameters for study groups at baseline and after 6 months.

Parameter		SRP Group	SRP + aPDT Group
**Subjects number (n)**		25	24
**Number of sites**		474	445
**Age (years) (mean** **±** **standard deviation)**		55.24 ± 3.41	55.58 ± 3.62
**Gender** **n (%)**	**Male**	14 (56.00%)	13 (54.16%)
	**Female**	11 (44.00%)	11 (45.84%)

**Table 2:** Clinical parameters for study groups at baseline and after 6 months.

Parameter	SRP Group (n = 25)			SRP + aPDT Group (n = 24)		
	Baselin *	+6 Month *	Δ ^#^	Baselin *	+6 Months *	Δ ^#^
**PI**	79.44 ± 6.31	17.72 ± 6.38 ^a^	62 (60–66)	80.04 ± 5.90	17.08 ± 5.14 ^a^	63 (60–67)
**BOP**	67.76 ± 6.57	8.08 ± 5.09 ^a^	60 (55–66)	68.67 ± 6.10	4.21 ± 3.85 ^ab^	65 ^c^ (55–69)
**PD (mm)**	5.54 ± 0.24	4.10 ± 0.22 ^a^	1.4 (1.4–1.5)	5.53 ± 0.24	3.56 ± 0.19 ^ab^	1.9 ^c^ (1.8–2.2)
**CAL (mm)**	4.51 ± 0.20	3.15 ± 0.17 ^a^	1.40 (1.20–1.50)	4.50 ± 0.22	2.58 ± 0.19 ^ab^	1.95 ^c^ (1.70–2.10)

Δ: level of decrease between evaluations; PI: Plaque Index; BOP: Bleeding on Probing Index; PD: probing depth; CAL: clinical attachment loss; * Values are expressed as Mean ± Standard Deviation; ^#^ Values are expressed as Median (Min–Max); ^a^ Intra-group *p* < 0.05 after 6 months (*t*-Test); ^b^ Inter-group *p* < 0.05 at the same time of evaluation (*t*-Test); ^c^ *p* < 0.001 (Man-Whitney test).

## Data Availability

The data used to support the findings of this study are available from the corresponding author upon reasonable request.
